# Reducing the Nighttime Fears of Young Children Through a Brief Parent-Delivered Treatment—Effectiveness of the Hungarian Version of *Uncle Lightfoot*

**DOI:** 10.1007/s10578-020-01103-4

**Published:** 2021-01-23

**Authors:** Krisztina Kopcsó, András Láng, Mary F. Coffman

**Affiliations:** 1 Department for Developmental and Clinical Psychology, University of Pécs, Ifjúság u. 6., Pecs, 7624 Hungary; 2 Hungarian Demographic Research Institute, Budapest, Hungary; 3 Independent Researcher, MN Rochester, USA

**Keywords:** Nighttime fears, Anxiety, Parent-delivered therapy, Bibliotherapy, Intervention

## Abstract

The aims of the present study were to evaluate the efficacy of a brief intervention, and to determine for whom the treatment works. 73 children between 3 and 8 years of age with significant nighttime fears were enrolled in an intervention group (n = 36) or in a waitlist group (n = 37). The intervention involved a 5-week parent delivered therapy. Assessments took place at baseline, post-treatment, and 20 weeks following baseline. In the intervention group, compared with the waitlist group, nighttime-related fears and phobic symptoms decreased more, whereas adaptive nighttime behavior increased to a greater extent. The more time children spent with exposure and relaxation games during the intervention, the more their separation anxiety and maladaptive nighttime behavior were reduced. Girls’ fear of darkness was reduced to a greater extent. The present study provides support for the use of parent-delivered therapy in the treatment of childhood nighttime fears.

## Introduction

Nighttime fears are common in children and are a normal part of child development [1–3]. “Nighttime fears are normal reactions to real or imagined threats at night” [1], usually defined as a heterogenous set of fears, including fear of imaginary creatures, burglars, animals, and other nighttime fears in addition to fear of the dark. According to previous results, 73.3% of children between 4 and 12 years of age report at least mild nighttime fears in a structured interview [3], confirming the high prevalence of such fears, while 20–30% of children are estimated to have severe nighttime fears [1]. Although nighttime fears do not constitute a separate diagnostic category, children with severe and permanent nighttime fears might meet the criteria for a specific phobia diagnosis [4]. An estimated 2% of children have darkness phobia [5], which mainly manifests itself by protests against going to bed and not wanting to sleep with the lights turned off [6]. Research findings suggest that nighttime fears or fear of the dark in childhood are associated with sleep problems [7, 8], co-sleeping with caregivers [9], externalizing and internalizing problems [7, 10–12], anxiety disorders [3], and fears, other than nighttime fears [11, 12].

Children with nighttime fears use a variety of coping strategies to reduce such fears [1, 3, 13]. Although they generally rate these coping behaviors as helpful [3], nighttime fears can become a permanent difficulty, requiring the assistance of a professional specialist. A possible reason for this may be the fact that young children frequently report seeking parental support to reduce their nighttime fears [3]. The presence and support of a parent in the process of falling asleep, however, can result in poorer sleep quality, and due to its rewarding nature, can contribute to maintaining nighttime fears [14–17]. In addition, the persistent avoidance of sleeping alone in the dark contributes in itself to maintaining pathological nighttime fears [18].

With regard to the treatment options, all currently supported interventions for early childhood anxiety entail exposure-based cognitive-behavioral therapy (CBT) with significant parental involvement [19]. With respect to the pre-treatment predictors of treatment response, Knight et al. [20] identified no consistent and clear pre-treatment predictors. Some predictors were, however, identified in more than one study, including primary anxiety diagnosis, severity, comorbidity and parental anxiety/psychopathology. Additional findings [21] supported the superiority of exposure-based treatments over alternative psychological approaches for specific phobia, and also showed that more sessions predicted more favorable outcomes.

CBT (with parental involvement) as the treatment of choice is not only effective and recommended, but also resource-intensive, and not available for many families who could benefit [22]. As a result, parent-delivered CBT has received considerable attention as a low-intensity, first-line treatment possibility [23]. Parent-delivered CBT, usually guided by written materials (self-help book, workbook, storybook) or a specialist, was found to be effective in the treatment of childhood anxiety [23–27].

For children’s nighttime fears, parent-delivered therapy, guided by the book *Uncle Lightfoot, Flip that Switch: Overcoming Fear of the Dark* [28, 29] proved to be an effective intervention in previous studies [30–32], that investigated its efficacy among children aged four to eight years, applying 4–5 weeks long intervention periods. Its therapeutic components, delivered at the child’s home, include in vivo exposure with phobic stimuli graduation, play, relaxation, verbal instigation, extinction, social and material reinforcement, symbolic modeling, cognitive modification, and parent training [32].

Despite the growing number of nighttime fear treatment studies, we know little about for whom and why certain treatments work [33]. In fact, none of the above studies [30–32] examined systematically the possible moderator variables. In addition, the samples of Lewis et al. [30] and Santacruz et al. [32] included children with specific phobia, while the severity was unclear in the studies of Mikulas et al. [31]. As a result, we cannot conclude whether the severity of nighttime fear plays an important role in the efficacy of *Uncle Lightfoot* or not.

In the present study, we sought to investigate the effectiveness of the Hungarian adaptation of the revised version of the second edition of *Uncle Lightfoot* [28] for young children with significant nighttime fears. Given prior studies, we hypothesized that due to the intervention, a significant decrease of nighttime fears’ severity and frequency will be detectable over time. Secondly, we anticipated an increase in adaptive nighttime behaviors and a decrease in separation anxiety in the intervention group. We also hypothesized that those changes will be significantly higher in the intervention group than in the waitlist group. Lastly, we aimed to identify the possible baseline- and intervention characteristics that have an effect on treatment efficacy.

## Method

### Design and Ethics

The study employed a two-arm, controlled trial design. Inclusion criteria for children were as follows: (1) should be between the ages of 3 and 8 years at the beginning of treatment, (2) should have a higher than average level of nighttime fears based on parental opinion, (3) should reach at least score five on a 10-point parent-reported visual-analogue scale (FOD), measuring the level of fear of the dark, (4) should not be involved in current treatment for nighttime fears or other internalizing problems, and (5) should be a native speaker of Hungarian. Thus, the basic criterion for inclusion was the subjective perception of the parent that the child was significantly afraid at nighttime. Contrary to previous efficacy studies, participation did not require a related clinical diagnosis.

Participating children were assigned to (1) a waitlist group; or (2) an intervention group that received 5-week parent-delivered bibliotherapy, using *Uncle Lightfoot*. Assignment was based on participants’ place of residence due to limited resources. (As recruitment and data collection was done solely by the first author, in-person meetings could only be carried out with families that were relatively easily accessible for her.) Variables were assessed at baseline, post-treatment and 20-week follow-up. The waitlist group received the self-help book after the follow-up measurement. The study received approval from the Hungarian United Ethical Review Committee for Research in Psychology (EPKEB) with the reference number 2017/62. Parents gave written informed consent and children gave assent to participate pre-treatment. Beyond the self-help book, participants received no compensation for their participation.

### Procedure

The recruitment of the participants occurred between March 2017 and June 2018 in Hungary, through advertisements in preschools, general health practitioners’ offices and on social networking sites, as well as by contacting psychologists working in preschools. Interested parents made contact via e-mail or telephone, then completed a short online questionnaire that assessed whether they met the inclusion criteria.

Parents from the waitlist group completed the baseline, post-treatment and follow-up questionnaires online. With them, no in-person contact was made, which made it possible to involve families living to a considerable distance from the first author who did data collection. They were informed that the aim of the assessment is to capture the changes or stability of their child’s nighttime fears during a 20-week-period. With families from the intervention group, two in-person sessions were scheduled at pre-treatment and post-treatment, while they completed the follow-up questionnaire online. They were informed that the aim of the assessment is to measure the efficacy of *Uncle Lightfoot*. Enrollment and personal interviews were conducted by the first author who is a psychologist.

The intervention consisted of the use of the Hungarian translation of the revised version of the second edition of *Uncle Lightfoot Flip that switch* [28]. *Uncle Lightfoot* is a self-help book, suitable for parent-delivered therapy of nighttime fears. After the study of Lewis et al. [30], the book was supplemented with two more chapters. The book was translated into Hungarian by a clinical psychologist who was also a teacher of English as a second language.

Beyond the story of a young boy who overcomes his fear of the dark, it offers games (cognitive behavioral interventions) that are designed to help children overcome their own nighttime fears. The recommended games are mainly in vivo exposures, such as finding toys or playing in the dark. Other recommended activities include therapeutic-elaborative drawing and relaxation elements.

The intervention was carried out by parents at home. Information about how to use *Uncle Lightfoot* was provided at the pre-treatment in-person session, as well as by the written parental guide in the book. Parents were instructed to read the book or play the recommended games on each evening during the five weeks, and to complete the book at least one time over that period. Parents completed a daily log, tracking their intervention activity.

### Participants

**Fig. 1:**
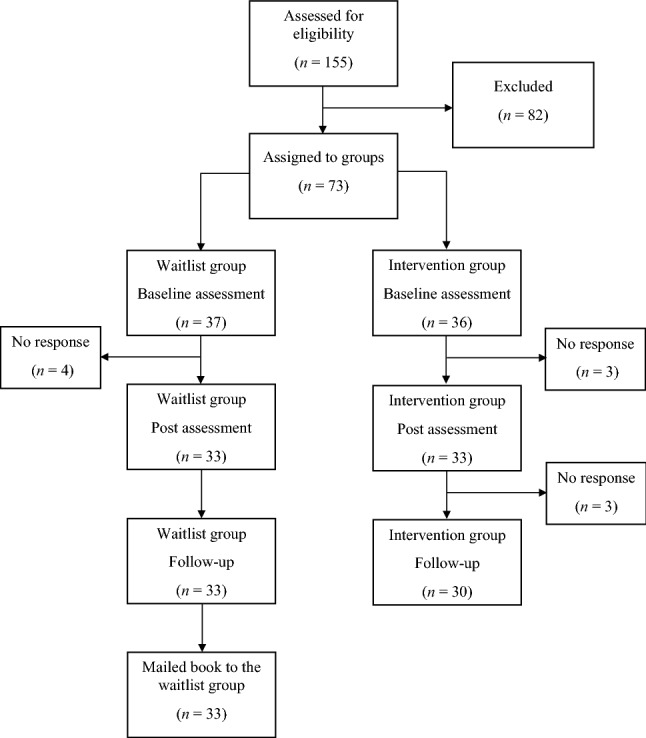
Flow of participants through each stage of the trial

Figure 1 describes the flow of participants through the study. From the 155 interested parents and their child, 82 did not meet the inclusion criteria or chose not to participate after being informed in detail. Of the included 73 families, 63 participated in assessments at all three time points.

The final sample consisted of 33 children in the waitlist group and 30 in the intervention group. Sociodemographic characteristics are presented in Table 1. The gender distribution was to a marginally significant extent unequal in the two groups. In addition, due to the group assignment method, parents in the intervention group were capital city residents or lived in Pest county near the capital, had to a marginally significant extent higher education, and were significantly older than parents in the waitlist group. No other differences were observed among the sociodemographic characteristics of the two groups.

**Table 1:** Sociodemographic characteristics of the final sample by test groups

	Intervention group (*n* = 30)	Waitlist group (*n* = 33)	*p*
Children			
Age *M* (*SD*) in months	64.53 (15.64)	65.12 (16.53)	0.885
Gender			
Female *n* (%)	16 (53.3)	10 (30.3)	0.064
Male *n* (%)	14 (46.7)	23 (69.7)	
Parent			
Age *M* (*SD*) in years	37.43 (4.67)	34.48 (4.37)	**0**.**012**
Gender			
Female *n* (%)	29 (96.7)	33 (100)	0.290
Male *n* (%)	1 (3.3)	0 (0)	
Number of children			
1 *n* (%)	7 (23.3)	5 (15.2)	0.329
2 *n* (%)	17 (56.7)	16 (48.5)	
3 or more *n* (%)	6 (20)	12 (36.4)	
Education level			
Higher education *n* (%)	24 (80)	18 (54.5)	0.085
Secondary *n* (%)	6 (20)	14 (42.4)	
Primary *n* (%)	0 (0)	1 (3)	
Subjective socioeconomic status			
Lower class *n* (%)	0 (0)	2 (6.1)	0.203
Lower-middle class *n* (%)	14 (46.7)	19 (57.6)	
Upper-middle class *n* (%)	16 (53.3)	12 (36.4)	

The *p*−values indicate the results of *t*−tests (in case of ages), and chi−square tests (in case of categorical variables). The *p*−value, indicating a statistically significant difference is in bold

### Measures

#### Child Interview

Children in the intervention group, with whom a personal meeting also took place, were asked to provide a rating of the level of their nighttime fear during the personal assessments at baseline and post-treatment. For that aim, a 3-point graphical assessment scale was used, which included faces, representing *no fear*, *mild fear* and *severe fear*.

#### Childhood Darkness Phobia Questionnaire (CDPQ) [34]

The CDPQ is a 5-item parent-report measure of children’s darkness or nighttime related fears of phobic intensity, including: (1) excessive fear triggered by nighttime or darkness, (2) the fear is persistent, occurring nearly every day, (3) the child avoids sleeping alone at nighttime in the dark, or endures it with anxiety, (4) the fear significantly impacts the child’s everyday life, and (5) the duration of fear lasts for several months. In such manner, the aspects taken into consideration by CDPQ, largely follow the DSM-5 (American Psychiatric Association [4]) criteria for specific phobia, although it does not require six months duration. Each item on the scale is answered on a 4-point scale (1 = *Not at all* to 4 = *Absolutely*). The total possible score on the CDPQ ranges from 5 to 20. A higher score indicates more pathological nighttime fears. The scale had acceptable internal consistency in the current study at post-treatment and follow up, while poor internal consistency at baseline (Cronbach’s Alphas [at baseline, post-treatment, follow-up, respectively] = 0.55, 0.72, 0.78).

#### What My Child Can Do At Night in the Dark (WICDAN-Parent Form) [35**]**

The WICDAN is an 11-item parent-report measure of children’s nighttime behaviors and self-efficacy in dealing with their fear of the dark. Each item on the scale is answered on a 3-point scale (0 = *No* to 2 = *Yes, easily*). The total possible score on the WICDAN ranges from 0 to 22. A higher score indicates more adequate nighttime behavior. In the study of Lewis et al. [30] the mean score (*SD*) of the WICDAN was 7 (3.6) among children with nighttime fears at baseline, while 16.75 (3.9) post-treatment and 16.25 (4.5) at one-month follow up. WICDAN was translated into Hungarian using the back-translation method. First, three psychologists independently translated the scale into Hungarian, and then agreed on a consensual version. Second, a Hungarian psychologist, working in England made a back translation of this version. Finally, this back-translated version was then reviewed and compared with the original English version by the developer of the scale and by the first author. A consensus about the final translation was reached without any further major modifications. The Hungarian translation had good internal consistency in the current study at each assessment (Cronbach’s Alphas [at baseline, post-treatment, follow-up, respectively] = 0.86, 0.81, 0.88). The measurement also included a 10-point (1 = *Not very afraid* to 10 = *Extremely afraid*) fear of the dark assessment scale (FOD).

#### Separation Anxiety Avoidance Inventory - Parent Version (SAAI-P) [36]

SAAI-P is a 12-item parent-report scale that measures to what extent the child avoids different relevant situations. Each item on the scale is answered on a 5-point scale (0 = *Never* to 4 = *Always*). SAAI-P was translated into Hungarian using the back-translation method. Three psychologists independently translated it into Hungarian, and agreed on a consensual version. Then, a Hungarian psychologist, working in England made a back translation of this version. Finally, this back-translated version was compared with the original English version by the first author who found it to be accurate. Due to the previous experience regarding age-related items [36], a *Not applicable* response option was added in the current study and an average of responded items was calculated and used in further analysis. Hence the possible score of separation anxiety ranges from 0 to 4, and a higher score indicates more frequent separation anxiety symptoms. The Hungarian version of the scale had good internal consistency in the current study at each assessment (Cronbach’s Alphas [at baseline, post-treatment, follow-up, respectively] = 0.83, 0.81, 0.81).

#### Intervention Log

Parents in the intervention group completed a daily report during the 5-week intervention, about: (1) the use of the book on the previous day (yes/no), (2) the duration of reading the book (in minutes), (3) the duration of playing games offered by the book (in minutes), (4) games applied and (5) difficulties experienced (if yes, please describe).

#### Treatment Evaluation

As part of the post-treatment assessment, parents in the intervention group evaluated the intervention, partly based on the *Treatment Evaluation Survey for Uncle Lightfoot, Flip That Switch-Revised*, developed by Coffman [37]. Information was collected regarding the (1) number of times the complete book was read to the child, (2) use of any reward or reinforcement, (3) use of the parent’s guide, (4) perceived effectiveness of the book and related satisfaction, (5) how much the child enjoyed *Uncle Lightfoot* in comparison to other books on a 5-point scale (1 = *Not really* to 5 = *Very much*). At follow-up, further use of *Uncle Lightfoot* after post-treatment assessment was reported.

### Data Analysis

Statistical analyses were performed using IBM SPSS Statistics 22 and criterion for statistical significances was set at the level of 5% (*p* < .05). Descriptive analyses are presented as proportions, means and standard deviations. For the continuous variables, comparisons were made through *t*-tests for independent samples, for the ordinal variables Mann-Whitney tests were used, and for the categorical ones, chi-square tests. Wilcoxon signed-rank tests were used to compare repeated ordinal measurements in the intervention group. Number of days and minutes spent reading *Uncle Lightfoot*, and number of minutes spent playing specific kind of games were summed up based on the Intervention Log for the whole intervention period, and used as continuous variables. Linear changes over time of continuous variables were analysed by mixed-design (split-plot) ANOVAs first, which were followed up by simple effect tests. Moderator and mediator variables regarding the intervention were first analysed by linear mixed effects models and then with Pearson’s correlations between the duration of CBT activities and change of certain variables over time.

## Results

### Treatment Retention

Total dropout rate was 13.7%: 20% in the intervention group and 12.12% in the waitlist group (*χ*2_(1)_ = 0.53, *p* = 0.467). Comparing children and their parents who completed each assessment to those who dropped out, no differences were observed among sociodemographic or clinical characteristics.

### Treatment Adherence and Evaluation

Parents in the intervention group were instructed to read the book and play the recommended games on each day during the five weeks if possible, and to read completely through the book at least one time over that period. According to the Intervention Log that was completely filled by 27 of the 30 parents, the book was read to the child 17 days on average over the 5 weeks (3.4 days per week on average), and the recommended games were played on ten days on average (2.1 days per week on average). On average, a total of 208 minutes was spent reading and 131 min was spent playing games from *Uncle Lightfoot*, though individual differences were remarkable (see Table 2). Most time was spent reading and playing exposure games.

**Table 2:** Dose of intervention based on the Intervention Log

	Min.	Max.	*M*	*SD*
Days of reading Uncle Lightfoot	5	30	17.19	6.58
Minutes of reading Uncle Lightfoot	75	585	207.52	110.09
Days of playing the games	0	29	10.30	8.11
Minutes of playing the games	0	470	131.33	120.57
Minutes of exposure game	0	350	101.67	90.70
Minutes of relaxation game	0	110	23.93	30.78
Minutes of elaborative drawing	0	45	5.37	10.91

*n* = 27

According to the post-treatment questionnaire, 20 parents (66.7%) read the book once with their child, three parents (10%) twice, two parents (6.7%) three times, three parents (10%) four or more times, and there were two parents (6.7%) who did not read through the book. Except for one parent, parental guide was read in part (14 parents, 46.67%) or in whole (15 parents, 50%). Reinforcement was used in almost half of the cases (14 [46.67%] parents). According to the follow-up questionnaire, 14 of the 30 families (46.67%) used the book further between post-treatment and follow-up, for an average of additional four weeks.

Overall, parents were satisfied with the book, based on the post-treatment questionnaire. From parents in the intervention group, 27 would recommend the book for other parents whose child is afraid at nighttime, while three would not recommend it. On a five-point scale, the mean satisfaction score with the book was 4.07 (SD = 0.98), and the average score of how much the child enjoyed its use compared to reading other books was 3.97 (SD = 1.30). 17 parents were sure that the book helped their child to reduce his/her nighttime fears, nine were uncertain about it and four parents thought that the book did not help. From those who said that the book helped or might have helped, 12 parents reported small, ten parents reported moderate, four parents reported great improvement, and one parent said that the child is not afraid any more.

Based on Spearman’s correlational analyses, the degree of the parent-reported improvement was moderately correlated with a decrease in phobic symptoms (CDPQ) from baseline to post-treatment assessment (r_s_ = 0.542, p = 0.003), with parental satisfaction (r_s_ = 0.674, p < 0.001), and with how much the child enjoyed the use of the book (r_s_ = 0.417, p = 0.022). Furthermore, higher parental post-treatment satisfaction was associated with higher intensity of the child’s baseline fear of darkness (FOD score, r_s_ = 0.370, p = 0.044), with higher decrease in fear of darkness (FOD) from baseline to post-treatment assessment (r_s_ = 0.334, p < 0.001), with lower adaptivity of his/her nighttime behavior at baseline (WICDAN, r_s_ = − 0.364, p = 0.048), and with the child’s higher enjoyment of the book (r_s_ = 0.722, p < 0.001).

### Characteristics of the Participants at Baseline

Based on the CDPQ, as indicated by *Rather yes* or *Absolutely* answers for all the five criteria of nighttime fear of phobic intensity, 23.8% of the participating children had clinical nighttime fears: 23.33% in the intervention group and 24.24% in the waitlist group (*χ*2_(1)_ = 0.01, *p* = 0.933).

At baseline no differences were observed among any measurements in the two groups. However, both at post-treatment and follow-up, children in the intervention group showed more adaptive nighttime behavior than children in the waitlist group, along with lower level of fear of the dark. In addition, they also had lower levels of phobic symptoms at follow-up. See Table 3 for descriptives and group comparisons at each assessment.

**Table 3:** Descriptives of variables by test groups and group comparison at each assessment

Variable	Baseline				Post-treatment				Follow-up			
	*M* (*SD*)				*M* (*SD*)				*M* (*SD*)			
	WG	IG	*p*	*t*	WG	IG	*p*	*t*	WG	IG	*p*	*t*
CDPQ	15.18 (2.37)	15.63 (2.55)	0.469	− 0.73	13.64 (3.32)	12.67 (2.80)	0.217	1.25	**13.64 (2.90)**	**10.47 (3.77)**	**< 0.001**	**3.76**
WICDAN	7.18 (3.66)	7.10 (4.25)	0.935	0.08	**8.39 (4.34)**	**11.77 (5.14)**	**0.006**	**− 2.82**	**9.39 (4.89)**	**13.97 (5.76)**	**0.001**	**− 3.41**
FOD	7.97 (1.29)	7.77 (1.43)	0.555	0.59	**7.46 (1.60)**	**5.87 (1.80)**	**< 0.001**	**3.71**	**6.61 (1.98)**	**5.17 (2.12)**	**0.007**	**2.79**
SAAI-P	2.12 (0.86)	1.97 (0.80)	0.475	0.72	2.14 (0.81)	1.77 (0.92)	0.090	1.72	1.72 (0.84)	1.44 (1.08)	0.242	1.18

Statistically significant differences are in bold

*WG* waitlist group; *IG* intervention group; *CDPQ* Childhood Darkness Phobia Questionnaire; *WICDAN* What My Child Can Do At Night in the Dark; *FOD* Fear of the Dark Assessment Scale; *SAAI−P* Separation Anxiety Avoidance Inventory − Parent Version

#### Changes in Child-Reported Fear of the Dark

Children in the intervention group self-rated their fear of the dark at baseline and post-treatment (*n* = 25) on a 3-point graphical assessment scale. Post-treatment scores (*Mdn* = 2, *M* = 2, *SD* = 0.76), compared to baseline scores (*Mdn* = 1, *M* = 1.52, *SD* = 0.59) were significantly lower (*Z* = − 2.68, *p* = 0.007).

### Changes in Parent Reported Fear and Anxiety

Changes of continuous variables over time were analysed by a mixed-design (split-plot) ANOVA at first, the results of which can be seen in Table 4. The main effect of time was significant in all cases, hence all the measured variables changed over time. The main effects of groups (waitlist or intervention), and the interactions between the effect of time and group were significant in all cases, except the variable separation anxiety.

**Table 4:** Changes in parent reported variables by test groups and time

Variable	df(error df)	*F*	*p*	Partial *η*^2^
CDPQ				
Main effect of time	2 (122)	**39.793**	**< 0.001**	0.395**
Main effect of group	1 (61)	**4.087**	**0.048**	**0.063**
Interaction	2 (122)	**11.313**	**< 0.001**	0.156*
WICDAN				
Main effect of time	2 (122)	**34.587**	**< 0.001**	0.362**
Main effect of group	1 (61)	**6.884**	**0.011**	**0.101**
Interaction	2 (122)	**9.526**	**< 0.001**	0.135*
FOD				
Main effect of time	1.57 (96.02)	**32.770**	**< 0.001**	0.349**
Main effect of group	1 (61)	**10.735**	**0.002**	0.150*
Interaction	1.57 (96.02)	**4.747**	**0.017**	**0.072**
SAAI-P				
Main effect of time	1.75 (106.97)	**9.651**	**< 0.001**	0.137*
Main effect of group	1 (61)	2.200	0.143	0.035
Interaction	1.75 (106.97)	0.502	0.583	0.008

Statistically significant differences are in bold

*CDPQ* Childhood Darkness Phobia Questionnaire; *WICDAN* What My Child Can Do At Night in the Dark; *FOD* Fear of the Dark Assessment Scale; *SAAI−P* Separation Anxiety Avoidance Inventory − Parent Version

*Moderate effect size (0.13 ≤ ηp2 < 0.26); **strong effect size (0.26 ≤ ηp2)

Thereafter, results of mixed design ANOVAs were followed up by simple effect tests based on the estimated marginal means. Overall results of simple effect tests, which are presented in Table 5, indicated that the intervention group showed greater improvements than the waitlist group. Pairwise comparisons, detailed in Table 6, clarified, that in case of the intervention group, the scores of WICDAN, CDPQ and FOD showed significant differences between each time points, while the scores of SAAI-P only showed a lower value at follow-up (compared to baseline and post-treatment). In case of the waitlist group however, fewer differences were detected. There were no differences between CDPQ scores at post-treatment and follow up, between FOD scores at baseline and post-treatment and between SAAI-P scores at baseline and post-treatment, while the score of WICDAN only differed between the baseline and follow-up.

**Table 5:** Multivariate simple effects of parent-reported variables within each level of time

Variable	*F*(2,60)	*p*	Partial *η*^2^
CDPQ			
WG	**7.337**	**0.001**	0.197*
IG	**46.237**	**< 0.001**	0.606**
WICDAN			
WG	**3.570**	**0.034**	**0.106**
IG	**33.132**	**< 0.001**	0.525**
FOD			
WG	**5.897**	**0.005**	0.164*
IG	**19.483**	**< 0.001**	0.394**
SAAI-P			
WG	**4.426**	**0.016**	**0.129**
IG	**3.970**	**0.024**	**0.117**

Statistically significant differences are in bold

*WG* waitlist group; *IG* intervention group; *CDPQ* Childhood Darkness Phobia Questionnaire; *WICDAN* What My Child Can Do At Night in the Dark; *FOD* Fear of the Dark Assessment Scale; *SAAI−P* Separation Anxiety Avoidance Inventory − Parent Version

*Moderate effect size (0.13 ≤ ηp2 < 0.26); **Strong effect size (0.26 ≤ ηp2)

**Table 6:** Pairwise comparisons of scale scores between each level of time by test groups

Scale	Group			Mean Difference	Std. Error	Sig	95% CI for Difference	
							Lower Bound	Upper Bound
WICDAN	WG	Baseline (t1)	Post-treatment (t2)	− 1.212	0.728	0.101	− 2.669	0.245
			**Follow-up (t3)**	− **2.212**	**0.823**	**0.009**	− **3.859**	− 0**.566**
		Post-treatment (t2)	Follow-up (t3)	− 1.000	0.737	0.180	− 2.473	0.473
	IG	Baseline (t1)	**Post-treatment (t2)**	− **4.667**	**0.764**	**0.000**	− **6.194**	− **3.139**
			**Follow-up (t3)**	− **6.867**	**0.864**	**0.000**	− **8.594**	− **5.140**
		Post-treatment (t2)	**Follow-up (t3)**	− **2.200**	**0.773**	**0.006**	− **3.745**	− **.655**
CDPQ	WG	Baseline (t1)	**Post-treatment (t2)**	**1.545**	**0.454**	**0.001**	**0.637**	**2.454**
			**Follow-up (t3)**	**1.545**	**0.543**	**0.006**	**0.459**	**2.632**
		Post-treatment (t2)	Follow-up (t3)	0.000	0.582	1.000	− 1.165	1.165
	IG	Baseline (t1)	**Post-treatment (t2)**	**2.967**	**0.476**	**0.000**	**2.014**	**3.919**
			**Follow-up (t3)**	**5.167**	**0.570**	**0.000**	**4.027**	**6.306**
		Post-treatment (t2)	**Follow-up (t3)**	**2.200**	**0.611**	**0.001**	**0.979**	**3.421**
FOD	WG	Baseline (t1)	Post-treatment (t2)	0.515	0.310	0.101	− 0.104	1.134
			**Follow-up (t3)**	**1.364**	**0.419**	**0.002**	**0.526**	**2.201**
		Post-treatment (t2)	**Follow-up (t3)**	**0.848**	**0.277**	**0.003**	**0.294**	**1.403**
	IG	Baseline (t1)	**Post-treatment (t2)**	**1.900**	**0.325**	**0.000**	**1.251**	**2.549**
			**Follow-up (t3)**	**2.600**	**0.439**	**0.000**	**1.722**	**3.478**
		Post-treatment (t2)	**Follow-up (t3)**	**0.700**	**0.291**	**0.019**	**0.119**	**1.281**
SAAI-P	WG	Baseline (t1)	Post-treatment (t2)	− 0.025	0.138	0.859	− 0.301	0.251
			**Follow-up (t3)**	**0.396**	**0.181**	**0.033**	**0.033**	**0.759**
		Post-treatment (t2)	**Follow-up (t3)**	**0.421**	**0.141**	**0.004**	**0.139**	**0.703**
	IG	Baseline (t1)	Post-treatment (t2)	0.198	0.145	0.176	− 0.091	0.488
			**Follow-up (t3)**	**0.531**	**0.190**	**0.007**	**0.150**	**0.911**
		Post-treatment (t2)	**Follow-up (t3)**	**0.332**	**0.148**	**0.028**	**0.037**	**0.628**

Statistically significant effects are in bold

*WG* waitlist group; *IG* intervention group; *WICDAN* What My Child Can Do At Night in the Dark; *CDPQ* Childhood Darkness Phobia Questionnaire; *FOD* Fear of the Dark Assessment Scale; *SAAI−P* Separation Anxiety Avoidance Inventory − Parent Version

### Moderator Variables

To identify for whom the intervention was effective, linear mixed effects models were applied at first. Due to the relatively small sample size of the intervention group, the significance of assumed fixed effects were examined in separate models, along with time (0, 5, 20 weeks). Models also included participating individuals as random effect.

Fixed effects on one hand included baseline variables: (1) child’s gender, (2) severity of fear of the dark, and (3) subjective-socioeconomic status of parents. On the other hand, fixed effects included specific characteristics of the intervention itself: (4) use of any reinforcement, (5) minutes of exposure exercises, (6) minutes of relaxation, and (7) how much did the child enjoy using the book. Outcome variables included the ones which showed changes over time, namely CDPQ, WICDAN, FOD, and SAAI-P. Detailed results of these linear mixed effects models are presented in Table 7.

**Table 7:** The effects of baseline and intervention characteristics on the changes of outcome variables

	Type III tests of fixed effects		Estimates of fixed effects				
	*F*	*p*	*t*	df	Estimate	95% CI	
						LB	UB
CDPQ							
Gender × time	0.28	0.598	− 0.53	81.67	− 0.03	− 0.13	0.08
Phobia × time	0.39	0.533	0.63	58	0.04	− 0.09	0.18
SES × time	0.38	0.539	0.62	81.68	0.03	− 0.07	0.13
Reinforcement × time	0.06	0.813	0.24	58	0.01	− 0.10	0.13
Exposure × time	2.79	0.101	− 1.67	52	> − 0.01	> − 0.01	> − 0.01
Relaxation × time	0.07	0.798	− 0.26	52	> − 0.01	> − 0.01	< 0.01
Joy × time	0.88	0.353	− 0.94	58	− 0.02	− 0.07	0.02
FOD							
Gender × time	**4.60**	**0.035**	**2.15**	**86.94**	**0.07**	**0.01**	**0.14**
Phobia × time	0.29	0.590	− 0.54	58	− 0.03	− 0.12	0.07
SES × time	0.99	0.324	0.99	86.95	0.03	− 0.03	0.10
Reinforcement × time	0.03	0.872	− 0.16	58	− 0.01	− 0.09	0.08
Exposure × time	0.59	0.447	− 0.77	52	> − 0.01	> − 0.01	< 0.01
Relaxation × time	< 0.01	0.997	> − 0.01	52	− 2.35	> − 0.01	< 0.01
Joy × time	2.34	0.132	− 1.53	58	− 0.02	− 0.06	0.01
WICDAN							
Gender × time	0.72	0.399	− 0.85	58	− 0.08	− 0.25	0.10
Phobia × time	0.20	0.659	− 0.44	58	− 0.05	− 0.25	0.16
SES × time	0.11	0.742	− 0.33	58	− 0.03	− 0.21	0.15
Reinforcement × time	< 0.01	0.960	− 0.05	58	> − 0.01	− 0.18	0.17
Exposure × time	4.01	0.050	2.00	52	< 0.01	− 1.72	< 0.01
Relaxation × time	**4.10**	**0.048**	**2.03**	**52**	**< 0.01**	**2.64**	**0.01**
Joy x time	0.32	0.576	0.56	58	0.02	− 0.05	0.09
SAAI-P							
Gender × time	0.20	0.660	− 0.44	77.03	− 0.01	− 0.03	0.02
Phobia × time	0.02	0.880	− 0.15	58	> − 0.01	− 0.04	0.03
SES × time	0.09	0.764	− 0.30	76.73	> − 0.01	− 0.03	0.02
Reinforcement × time	0.09	0.771	− 0.29	58	− 0.01	− 0.04	0.03
Exposure × time	**12.25**	**0.001**	**− 3.50**	**52**	**> − 0.01**	**> − 0.01**	**> − 0.01**
Relaxation × time	**4.27**	**0.044**	**− 2.07**	**52**	**> − 0.01**	**> − 0.01**	**− 1.58**
Joy × time	0.15	0.697	− 0.39	58	> − 0.01	− 0.01	0.01

Statistically significant effects are in bold

*CDPQ* Childhood Darkness Phobia Questionnaire; *FOD* Fear of the Dark Assessment Scale; *WICDAN* What My Child Can Do At Night in the Dark; *SAAI−P* Separation Anxiety Avoidance Inventory − Parent Version

Child’s gender affected the reduction of the level of fear of the dark (FOD). Namely, girls’ fear of darkness was reduced to a greater extent during the 20-week period than that of boys. Whether the child had a nighttime fear that reaches the intensity level of a phobia at the start of the study had no effect on the temporal change of any of the tested outcome variables, nor did the level of the subjective socioeconomic status of the parent.

The use of reinforcement did not affect the changes of the outcome variables. However, the time spent with exposure and relaxation games contributed to the temporal changes in the adaptivity of nighttime behavior and separation anxiety.

As a next step, correlations between the duration of recommended activities, and temporal changes in separation anxiety and nighttime behavior adaptivity were analyzed to further understand the moderating effect. These analyses suggested that the more time a child spent with exposure games, the greater was the reduction in separation anxiety for post-treatment (*r* = 0.525, *p* = 0.005) and follow-up (*r* = 0.549, *p* = 0.003), as well as the greater was the increase of nighttime behavior adaptivity at post-treatment (*r* = 0.610, *p* = 0.001) and follow-up (*r* = 0.519, *p* = 0.006). Furthermore, the more time spent with relaxation games, the greater was the reduction in separation anxiety for post-treatment (*r* = 0.384, *p* = 0.048), and the greater was the increase of nighttime behavior adaptivity at post-treatment (*r* = 0.389, *p* = 0.045) and follow-up (*r* = 0.462, *p* = 0.015). How much the child enjoyed using the book compared to other books did not influence the change of any outcome variables.

## Discussion

The aim of the present study was to evaluate the efficacy of a parent-delivered cognitive-behavioral therapy among three to eight years old children, guided by a self-help book. Further, we wanted to determine for whom and why the treatment works. As opposed to previous efficacy studies [30, 32], the study included children with non-clinical problems as well. According to parent-reported presence of phobic symptoms (excessive and frequent fear, avoidance or anxiety, impact on everyday life, several months duration), only 24% of the participating children had clinical nighttime fears: 23% in the intervention group and 24% in the waitlist group. Despite differences in selection criteria, the present study also found empirical evidence for the efficacy of parent-delivered CBT, guided by *Uncle Lightfoot*, which is in line with previous research [30–32].

The total dropout rate was 13.7, which is consistent with previous research. Parents in the intervention group were asked to read the book and play the recommended games on each day during the five weeks if possible, and to complete the book at least once. In their subjective evaluation, parents were generally satisfied with the book, and 57% of them were sure that it helped their child to become less fearful. According to the Intervention Log, parents used the book on an average of seventeen days (out of the 35 days available), thus, an average of 3.4 days per week, and typically did not follow the daily use instruction. However, most of them (93%) completed the book at least once, and there were eight parents (27%) who read it at least two times. Thus, in the present study, treatment dosage was lower than in the study of Lewis et al. [30] in which nine children participated, who read the book on average 4.6 days a week during a four-week intervention period, and six out of nine parents (67%) read it at least two times. It can be assumed, that similar to the findings of Rapee et al. [26], a standard CBT treatment with a therapist would result in greater change than a parent-delivered CBT, partly due to a higher dosage of treatment. The comparison of the present intervention with a therapist-delivered CBT is difficult though, since CBTs usually take longer duration than a few weeks. Therefore, it would be necessary to test the efficacy of *Uncle Lightfoot* in further studies over an 8 to 16 weeks period at a higher dosage.

Nevertheless, based on the changes in the scores of parent-reported scales over time, the present intervention effectively reduced children’s nighttime fears, darkness phobia symptoms, and increased the adaptivity of their nighttime behaviors, compared to a waitlist control group. Improvements in the intervention group was significant for the post-treatment assessment and became even more pronounced for the follow-up assessment. Furthermore, children themselves in the intervention group rated their nighttime fears to be lower at post-treatment compared to baseline. On the other hand, the waitlist group also showed some, though smaller, improvements in nighttime fears, and the two groups showed similar improvements in separation anxiety. With regards to separation anxiety, it should be emphasized, that the separation anxiety of children in the intervention group was not particularly high at baseline, based on their SAAI-P score (M = 1.97, SD = 0.80), compared to previous mean scores which were 1.81 (SD = 0.72) for a school sample, 3.52 (SD = 0.89) for children with separation anxiety disorder, and 2.91 (SD = 1.03) for children with other anxiety disorders [36]. Previous efficacy studies related to *Uncle Lightfoot* treated the issue of separation anxiety differently. In the study of Santacruz et al. [32] separation anxiety was an exclusion criterion, while in the study of Lewis et al. [30] the change in separation anxiety was assessed, and showed a declining trend, similarly to the present study. There was, however, no control group in the study of Lewis et al. [30] for which the improvement could be measured, thus it would be interesting to include children with high levels of separation anxiety in future *Uncle Lightfoot*-related controlled trials. In point of fact, in a recent study of Rafihi-Ferreira et al. [38], including children who had separation anxiety and co-slept with their parents, a brief parent-delivered intervention (CBT-based bibliotherapy plus doll) was superior to a waitlist condition, by reducing sleep problems, co-sleeping, separation anxiety, general anxiety and fears, behavior problems and nighttime fears.

Regarding moderator variables, the Uncle Lightfoot intervention was more effective in reducing separation anxiety in children and increasing adaptivity of their nighttime behavior when families spent more time with exposure and relaxation games, which proves the importance of the dose of treatment [21, 26]. Although the use of reinforcement did not affect treatment efficacy in this study, previous research [31, 39] suggested that tangible rewards might be of benefit based on one very small study (n = 12). Future studies would be useful to explore this issue further.

Efficacy was not consistently influenced by demographic and clinical factors such as subjective socioeconomic status of the parents, age of the child, or presence of nighttime fear that reaches the intensity level of a phobia, which is in line with previous results [20]. Girls, however, showed greater improvements on one of the scales in the present study. This result may be connected to the fact, that girls tend to seek parental support more than boys [3], and presumably due to that, the decline in nighttime fears—and related behaviors—is more noticeable in the case of girls, which may bias the answer on a simple, one-item subjective parental scale. How much the child enjoyed using the book compared to other books did not influence treatment effects; however, it was highly associated with parental satisfaction.

One fundamental limitation of the present research is that it did not include a placebo control group, which makes it inadequate to assess the impact of non-specific elements of the intervention (e.g., active participation in a process, believed to reduce nighttime fears; more time spent with the child as a result of following intervention instructions). In addition, personal contact was made with the intervention group only, the therapeutic effect of which cannot be excluded [25]. Generalizability of our results is also limited, because the study did not include parents with lower socio-economic status or education. In addition, assignment to groups was not randomized. Though the location-based assignment did not appear to cause remarkable bias in the results, as the two groups did not differ in relevant demographic or any psychological variables at baseline, the impact of the slight demographic differences between the groups on the results cannot be excluded.

## Summary

Nighttime fears are frequent among children, and are often related to internalizing and externalizing problems and sleep disorders. The aims of the present study were to evaluate the efficacy of a brief parent-delivered intervention, guided by a self-help book, and to determine for whom the treatment works. 73 children between three and eight years of age with significant nighttime fears were enrolled in an intervention group (n = 36) or in a waitlist group (n = 37), based on participants’ place of residence. The intervention involved parents reading the Hungarian translation of *Uncle Lightfoot, Flip that Switch: Overcoming Fear of the Dark* [28] with their children for 5 weeks while engaging in recommended activities. Assessments took place at baseline, post-treatment (i.e., 5 weeks following baseline), and 20 weeks following baseline. Intervention activities were daily administered by parents during treatment. In the intervention group, compared to the waitlist group, nighttime-related fears and phobic symptoms decreased more, whereas adaptive nighttime behavior increased to a greater extent. The more time children spent with exposure and relaxation games during the intervention, the more their separation anxiety and maladaptive nighttime behavior were reduced. Girls’ fear of darkness was reduced to a greater extent. Based on the results, the use of the Hungarian version of *Uncle Lightfoot* could be an effective first-line, low-intensity treatment option for children with nighttime fears, before recommending more intensive, expensive and sometimes less accessible professional treatment options. Future studies should assess whether using *Uncle Lightfoot* for a longer period would be more efficient and whether children with separation anxiety could also benefit from using it.
